# Splice Variants of Na_V_1.7 Sodium Channels Have Distinct β Subunit-Dependent Biophysical Properties

**DOI:** 10.1371/journal.pone.0041750

**Published:** 2012-07-24

**Authors:** Clare Farmer, James J. Cox, E. V. Fletcher, C. Geoffrey Woods, John N. Wood, Stephanie Schorge

**Affiliations:** 1 UCL Institute of Neurology, Queen Square, London, United Kingdom; 2 UCL Wolfson Institute of Biomedical Research, London, United Kingdom; 3 Department of Medical Genetics, Cambridge Institute for Medical Research, Addenbrooke’s Hospital, Cambridge, United Kingdom; University of Bristol, United Kingdom

## Abstract

Genes encoding the α subunits of neuronal sodium channels have evolutionarily conserved sites of alternative splicing but no functional differences have been attributed to the splice variants. Here, using Na_V_1.7 as an exemplar, we show that the sodium channel isoforms are functionally distinct when co-expressed with β subunits. The gene, *SCN9A,* encodes the α subunit of the Na_V_1.7 channel, and contains both sites of alternative splicing that are highly conserved. In conditions where the intrinsic properties of the Na_V_1.7 splice variants were similar when expressed alone, co-expression of β1 subunits had different effects on channel availability that were determined by splicing at either site in the α subunit. While the identity of exon 5 determined the degree to which β1 subunits altered voltage-dependence of activation (*P* = 0.027), the length of exon 11 regulated how far β1 subunits depolarised voltage-dependence of inactivation (*P* = 0.00012). The results could have a significant impact on channel availability, for example with the long version of exon 11, the co-expression of β1 subunits could lead to nearly twice as large an increase in channel availability compared to channels containing the short version. Our data suggest that splicing can change the way that Na_V_ channels interact with β subunits. Because splicing is conserved, its unexpected role in regulating the functional impact of β subunits may apply to multiple voltage-gated sodium channels, and the full repertoire of β subunit function may depend on splicing in α subunits.

## Introduction

**Figure 1:**
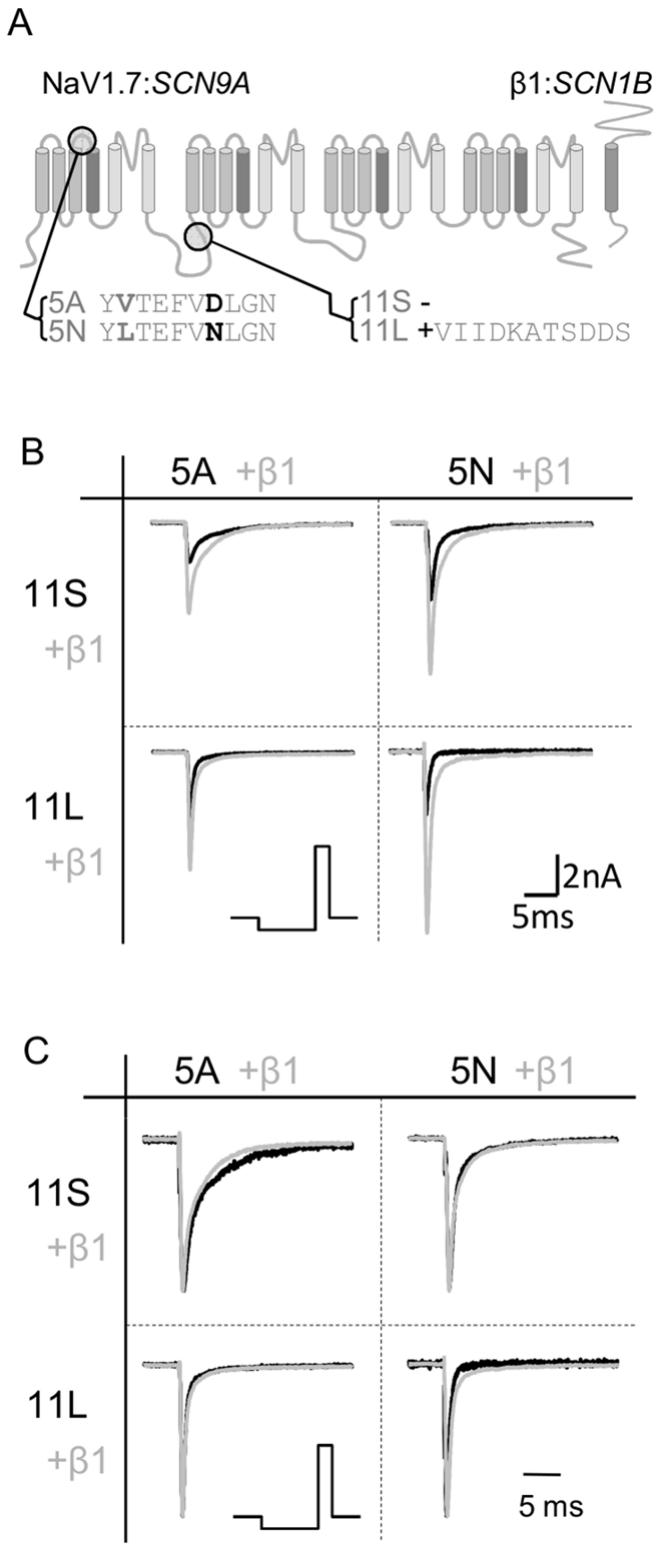
Overview of sites of splicing and channel behaviour. (A) A schematic of the α subunit of the Na_V_1.7 channel with the location of the two changes introduced by splicing indicated. (B) Example traces from each of the splice variants in the absence (dark lines) and presence (grey lines) of β1 subunits. Currents were elicited by a voltage step to −10 mV from a 100 ms prepulse to −120 mV to remove inactivation (protocol shown below). (C) Traces from (B) scaled to show a lack of effect of β1 on the kinetics of α subunit inactivation and persistent current.

As with other neuronal TTX-sensitive sodium channels, *SCN9A* is subject to alternative splicing at least at two sites. The gene contains two alternate mutually exclusive exons (5A and 5N) encoding the extracellular linker and voltage-sensor in the first domain, a feature shared with most neuronal sodium channels (Figure 1A; [1]). Along with other sodium channels [2], [3], [4], splicing of exon 5 is developmentally regulated in *SCN9A* [5]. The second conserved splicing site is an alternate recognition sequence at the end of exon 11 that allows a short (11 S) or long (11 L) intracellular linker to be produced between domains I and II of the channel (Figure 1A; [6], [7]). Again there is a developmental change in expression with adult dorsal root ganglion (DRG) neurons showing increased levels of 11L compared to neonatal neurons [5]. Splicing at exon 11 has recently been shown to alter the sensitivity of the channel to phosphorylation [6], suggesting that splicing may modulate the sensitivity of the channel to intracellular regulation, but the interaction between the splice variants and accessory subunits has not been investigated.

Sodium channels are thought to be composed of multiple subunits, with β subunits modulating the expression, localisation and gating of the channels. β subunits are already recognized as potential therapeutic targets [8], but it is unclear what determines their assembly with different α subunits. The dynamic regulation of β subunits during development [9] and diseases such as epilepsy [10], suggests that these subunits may not be constitutively present in sodium channel complexes. Moreover the different regulation of splicing and β subunit expression in development and disease suggests that different splice variants may associate with different β subunits.

The voltage-gated sodium channel Na_V_1.7 plays an important role in nociception in peripheral sensory neurons [11], [12]. Changes in the activity of the channel can have striking and specific effects on sensitivity to pain. Homozygous loss of function mutations in *SCN9A*, the gene which encodes the α subunit of Na_V_1.7, is associated with inability to feel pain [13], while mutations that increase channel function lead to disorders characterised by extreme pain [14], [15], [16]. Because of its importance in pain, there is potential clinical relevance for factors that modify the activity of Na_V_1.7 channels. Two possible factors which could modulate the channel during development and disease are alternative splicing and assembly with accessory subunits.

We asked whether different splice variants could interact differently with a β subunit to modify the gating of Na_V_1.7 channels. As proof of principle we have focused on β1 subunits, which are not covalently linked to α subunits, and which are downregulated in some neurological disorders [10], [17]. Our data suggest that β1 subunits interacting with different α subunit splice variants could provide meta-regulation of channel gating.

## Methods

### Constructs

The four *SCN9A* constructs (5A11S, 5N11S, 5A11L, 5N11L) were cloned using standard molecular techniques and comprised the *SCN9A* clone (NM_002977) in a modified pcDNA3 vector followed by a polio IRES-DsRed2 fragment which permitted identification of transfected cells. Human sodium channel β1 was amplified from human brain total RNA and cloned into the TOPO-directional vector (Invitrogen). All primer sequences are available upon request.

### Cell Transfection

HEK293 (ATCC) cells were transfected with *SCN9A* constructs using lipofectamine 2000 (Invitrogen) and plated onto poly-d-lysine coated coverslips. All recordings were done 24–72 hours after transfection. Cells expressing Na_v_1.7 were identified using red fluorescence, and cells co-expressing β1 and GFP showed both red and green fluorescence. The amount of *SCN9A* DNA transfected remained constant throughout all experiments and when β1 and GFP (pTracer, Invitrogen) were cotransfected it was in a ratio of 3∶1∶1 (SCN9A:β1:GFP).

### Electrophysiology

Whole-cell voltage clamp recordings were performed using an Axopatch 200 B amplifier at room temperature using standard techniques. Extracellular solution comprised (mM) NaCl 145, KCl 4, CaCl_2_ 1.8, MgCl_2_ 1, HEPES 10, (pH 7.35 with NaOH); intracellular (mM) CsCl 150, EGTA 10, HEPES 10, (pH 7.35 with CsOH). Data was sampled at 50 kHz, filtered at 5 kHz and leak currents were subtracted using a P/4 protocol. Average series resistance was 5.1±0.3 MΩ and was compensated by 75–90%. Tau of inactivation was measured by fitting a standard exponential function to the decaying current and persistent current was determined by measuring the current 20 ms after the start of the voltage step and expressing it as a percentage of the transient current in the same step. To assess voltage dependence of activation current-voltage families were obtained and peak sodium currents measured at each voltage. Channel conductance was calculated using the formula G_Na_ = I/(V-Vrev) where G_Na_ is sodium conductance, I is peak current, V is the voltage step and Vrev is the measured reversal potential which was determined for each cell by fitting a straight line through the linear portion of the current-voltage relationship. Sodium conductances were normalized and fitted with a standard Boltzmann function: G_Na_/G_Na_max = 1/(1+ exp((V_50_-V)/k)) where G_Na_/G_Na_max is the normalized conductance, V_50_ is the voltage which produces half maximal conductance and k is the slope factor. To reduce the risk of voltage clamp error in the estimates, cells with activation slopes less than 4 were discarded from the analysis. The protocol used to assess the voltage dependence of inactivation produced biphasic inactivation curves in a number of cells (37/59). The curves consisted of a large and stable negative component and a smaller, more variable positive component. The latter reflects a proportion of Na_v_1.7-mediated current which is not fully inactivating, probably due to variations in the development of closed-state inactivation which can be slow in these channels [18]. In these cells a double Boltzmann curve was fitted using the following function: I_Na_  =  A/(1+ exp((C−V/K)) + (1−A)/(1+exp((D−V)/L)) where A is a scaling factor, C and D are V_50_ values and K and L are slope factors of the two components of the fit. To improve the quality of the fit D and L (V_50_ and slope of minor component) were fixed. This equation was manually entered into the GraphPad Prism non-linear curve fitting software. The V_50_ values for the more negative component were combined with the V_50_ values obtained for single Boltzmann fits from the remaining cells in order to compare the voltage dependence of inactivation between splice variants. Data was acquired and analysed using Labview software with programs written in house (DM Kullmann, UCL). Additional data analysis and statistical testing was performed using Clampfit, Microsoft Excel, Microcal Origin Pro, and GraphPad Prism software. All data is presented as mean ± SEM.

## Results

In individual neurons, multiple sodium channel α and β subunits may be co-expressed, and each α subunit may be present as a mixture of splice variants. In order to determine whether the β subunits had specific interactions with different splice variants of an α subunit, we used a line of HEK293 cells, which we have previously shown to have negligible expression of endogenous α and β channel subunits [19]. At physiological temperatures, we have found that the modulation imposed by β subunits is obscured, and because we were interested in whether it is possible for alternative splicing in the α subunit to alter the effects of β subunit co-expression, we carried out recordings at room temperature where interactions are more apparent. Our goal was to test the hypothesis that alternative splicing in α subunits could change the gating properties affected by β subunit expression.

When expressed alone in HEK293 cells, all human Na_v_1.7 splice variants produced comparable sodium currents, with no significant difference between the variants in peak current density, rate of inactivation, or persistent currents (for each parameter, P>0.1; 1-way ANOVA; Figure 1; Table 1). When held at −80 mV the inactivation curves of the Na_v_1.7 variants were characterised by a significant second component, representing 18±2% of the total current, which inactivated at more depolarised potentials (approximately −30 mV). We confirmed that when recording using intracellular solutions containing CsF, this component was suppressed (data not shown), suggesting it may be in part due to intracellular signalling. In order to improve the fit to the main component of inactivation when using solutions that did not contain fluoride, the V_50_ and slope of this second component were fixed, and only the fraction was allowed to vary. In all variants and subunit combinations, the percentage of current mediated by this component was similar (P = 0.39; 1-way ANOVA).

**Table 1:** Macroscopic gating properties of *SCN9A* splice variants with and without β1 subunits.

cDNAs	*n*	INaT (pA/pF)	INaP (%INaT)	Tau_inac_ (ms)
5A11S	*6*	−105±50	3.7±2.0	1.15±0.22
+β1	*6*	−260±58 ‡	3.3±1.0	1.78±0.50
5N11S	*10*	−250±64	2.7±0.6	0.97±0.15
+β1	*7*	−362±83 ‡	2.9±0.6	1.13±0.33
5A11L	*9*	−207±42	3.0±1.1	0.99±0.25
+β1	*7*	−321±67 ‡	1.9±0.3	0.78±0.14
5N11L	*6*	−151±26	2.7±1.0	0.89±0.32
+β1	*8*	−453±80 ‡	1.9±0.5	0.67±0.09

‡ values with β1 are significantly larger than without after 2-way ANOVA.

Co-expression with the β1 subunit consistently increased the mean peak currents, and this was highly significant overall (P = 0.00057, 2-Way ANOVA), with no significant differences between the current densities driven by splicing (P = 0.20 all 4 variants, 2-Way ANOVA). In contrast, the co-expression of β1 subunits had no overall effect on the rate of inactivation or persistent current for the different splice variants (Table 1, P>0.1, One-way ANOVA).

**Figure 2:**
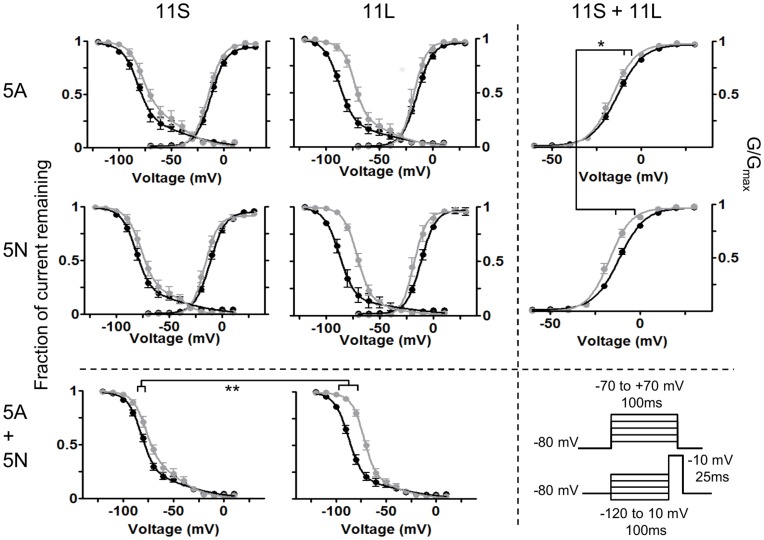
Exon 5 is preferentially coupled with activation and exon 11 modifies inactivation. Black lines are *SCN9A* alone, and grey lines are with β1 subunits. The columns are arranged according to the length of exon 11, and rows by identity of exon 5. The bottom row shows pooled data from each exon 5 variant (irrespective to exon 11 length). The right column shows pooled data for each form of exon 11 (independent of identity of exon 5). Numbers of cells are as in the table. Voltage protocols are shown for activation (top) and inactivation (bottom).

The β1 subunits induced a slight hyperpolarizing shift in the voltage dependence of activation for all four splice variants, with the two variants containing the 5 N exon tending to larger shifts, independent of which exon 11 splice form was present (Figure 2, Table 2). Voltage dependence of inactivation was also altered, with the β1 subunits producing a depolarising shift in the inactivation curves. However, in this case the identity of exon 11 appeared the driving factor, with variants containing exon 11 L having a larger shift than those containing exon 11 S, and the identity of exon 5 not appearing to alter inactivation (Table 2). Thus co-expression of β1 subunits may combine with splicing in different domains of the channels to selectively alter different parameters of channel gating.

**Table 2:** Voltage dependent parameters of *SCN9A* splice variants with and without β1 subunits.

		Activation		Inactivation	
cDNAs	*n*	V_50_	Slope	V_50_	Slope
5A11S	*15*	−11.0±1.0	7.5±0.4	−83.0±1.4	−7.1±0.5
+β1	*12*	−13.1±1.4	6.9±0.4	−78.3±2.8	−5.6±0.5
5N11S	*13*	−10.0±0.7	8.0±0.5	−81.5±1.3	−6.5±0.3
+β1	*10*	−15.8±1.8 *	6.1±0.6	−76.8±2.2	−6.0±0.4
5A11L	*9*	−14.4±1.5	7.0±0.4	−87.3±1.4	−6.8±0.1
+β1	*7*	−12.4±1.2	6.1±0.4	−74.1±1.3 **	−5.8±0.7
5N11L	*7*	−10.5±0.6	7.3±0.5	−87.3±1.8	−7.0±0.5
+β1	*7*	−14.6±2.4	6.2±0.6	−71.5±2.0 **	−5.5±0.1

* p<0.05, **p<0.001 compared to same splice variant without β1 after 1-way ANOVA with Tukey-Kramer Multiple Comparisons Test.

We hypothesized that the effects of exon 5 on activation could be uncoupled from the effects of exon 11 on inactivation, and to test this we pooled data from exon 11 L and 11 S cells to ascertain the effects of exon 5 on activation, and pooled the data from exon 5A and 5N cells to isolate the effects of exon 11 on inactivation.

Pooling cells according to exon 5 revealed that β1 co-expression produced a larger hyperpolarising shift in activation for those containing 5N compared to 5A, regardless of exon 11 background (Figure 2, right column). While co-expression of β1 shifted the activation of channels containing exon 5N an average of 5.1 mV (from −10.2±0.5 to −15.3±1.4 mV) it only changed the activation of channels containing 5A on average by less than 1 mV (0.8 mV; from −12.1±0.9 mV to −12.9±1.0). In total, the shift imposed by β1 on 5N-containing channels was significantly larger than that on 5A-containing channels (p(Δ5N = Δ5A) = 0.027; 2-way ANOVA).

The effects on inactivation were more pronounced, and more closely driven by the length of exon 11. The β1-mediated depolarising shift in inactivation, when pooled according to exon 11 variant, showed that cells with the long variant had a much larger shift than cells containing the short variant, regardless of the identity of exon 5 (Figure 2, bottom row). While the presence of β1 depolarised the inactivation of channels with the short exon by an average of 4.5 mV (from −82.1±0.9 to −77.5±1.7 mV) the shift in inactivation of channels containing exon 11 L was greater than 10 mV, or an average of 14.6 mV (from −87.2±1.1 to −72.7±1.2 mV). The change was significantly much larger in 11 L channels than those containing 11 S (p(Δ11 L = Δ11 S) = 0.00012; 2-way ANOVA).

**Figure 3:**
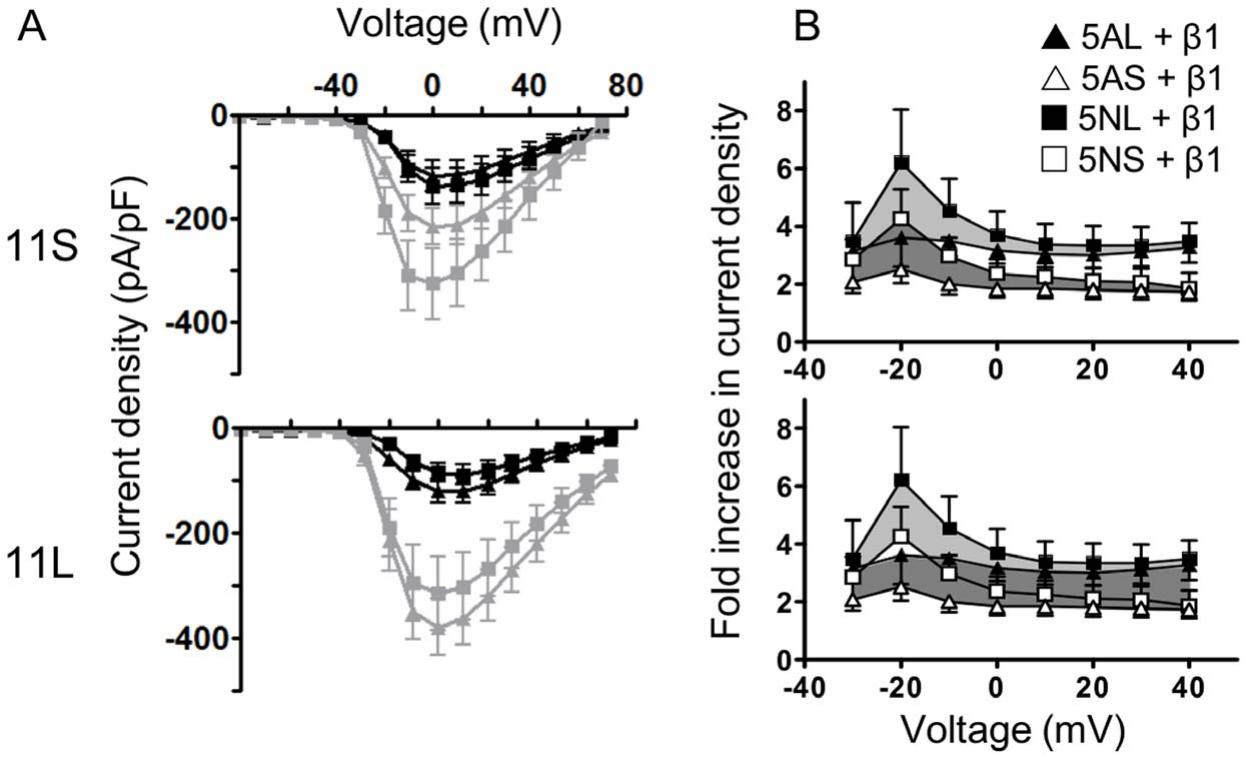
β1 subunits have different effects on current densities depending on splicing in *SCN9A*. (A) Un-normalised current density plots showing increased current due to expression of β1 subunits. Triangles  =  exon 5A, Squares  =  exon 5N. Grey  =  with β1 subunits. To assess the impact of the β subunits we held cells at physiological potentials (−80 mV) and stepped to a range of potentials (−80 to +70 mV). (B) Consequences of β1 subunit co-expression for steps to different potentials. The same data are shown in top and bottom panels, but in the top panel the greyed-out triangular area indicates the difference in current amplitude between variants that differ at exon 5, while in the bottom panel the shaded areas indicate the difference introduced by changing the length of exon 11. In contrast to the changes due to exon 5, which are much larger at −20 mV than at most other potentials, the changes imposed by exon 11 are of similar amplitude over the range of potentials tested. Exon 11 length (bottom panel) is associated with approximately a two fold increase of current density over all voltages. The identity of exon 5 induces a specific increase (∼2 fold) in the neighbourhood of −20 mV (top panel), but virtually no difference at more depolarised potentials. Cell numbers are as in Table 2.

The shift in inactivation introduced by β1 subunits in the presence of exon 11 L introduced potentially important changes in channel availability near typical resting membrane potentials. For example, variant 5N11L changes from more than 60% inactivated at −80 mV in the absence of β1 subunits to less than 20% inactive when β1 is co-expressed, indicating that the presence of β1 alters the availability of nearly half the channels at physiological resting potentials. However a direct prediction of channel availability during voltage steps may be confounded by a changing voltage dependence of activation, such as that caused by splicing of exon 5, or by altered trafficking in the presence of β1 subunits. We therefore measured the current densities generated by the different splice variants over a range of potentials in the presence and absence of β1 subunits when cells were held near physiological resting potential (Figure 3).

When current densities for the different variants were systematically compared over a range of potentials, the different splice variants revealed patterns of increased current density that were consistent with exon 5 targeting activation and exon 11 altering availability by shifting steady state inactivation. Variants with the ‘long’ version of exon 11, had larger increases than 11 S variants over a range of strongly depolarized potentials (0 to +40 mV, Figure 3B bottom), suggesting that the change in inactivation had a significant effect on channel availability when stepped from −80 mV to a range of potentials. The splicing at exon 5, in contrast, altered the amplitude of currents at more hyperpolarized potentials (−40 to −20 mV, Figure 3B, top), with no effect on the strongly depolarized currents (0 mV and above), which is consistent with the larger effect on activation and little effect on channel availability at the initial −80 mV holding potential. The combination of splicing and β1 subunits meant that the largest shift, given by exon 5 N combined with the long form of exon 11 gave over a 6 fold increase in current density at −20 mV, while the opposite combination of exon 5A with short exon 11 gave just over 2-fold increase at the same potential.

Taken together, our results suggest that β1 subunits interact differently with Na_V_1.7 channels encoded by different splice variants of *SCN9A* and can differentially regulate channel availability at resting potentials and during activation with large effects on channel availability and activation.

## Discussion

### Meta-regulation of Sodium Channels by β Subunits and Alternative Splicing

Our data reveal that splicing at two sites within a sodium channel α subunit can change how β1 subunits modulate the function of the channels. In the first domain of the α subunit, alternative splicing of exon 5, which changes the S3–S4 linker, modulates the effect of β1 subunits on the voltage-dependence of activation. In contrast, the length of exon 11, which changes the first intracellular loop of the channel, determines the degree to which β1 subunits shift the voltage-dependence of steady-state inactivation of the channels. These data suggest that the functional effects of splice variants can be modified by accessory subunits, and because alternative splicing in neuronal sodium channels is highly conserved [1], it is possible that similar interactions between splicing and accessory subunits may occur with other Na_V_ channels. Our data also indicate that the repertoire of β subunit functions can be expanded by considering their potential ability to discriminate between α subunit splice variants.

Our observations are consistent with work from other groups suggesting that in the presence of β1 subunits there is little difference between macroscopic currents from *SCN9A* splice variants recorded from HEK cells held at strongly hyperpolarized potentials [6]. However, recording at more physiological potentials and varying accessory subunit expression reveals significant differences between the behaviour of splice variants. These data suggest a meta-regulation – a second level of regulation of channel function – can be produced by the distinct interaction of different splice variants with with accessory subunits, and provide a new insight into how alternative splicing may modify the function of sodium channels. Because alternative splicing of sodium channels has been shown to be highly conserved [1], the four α subunit splice variants and one accessory subunit, *SCN1B*, that we have investigated here represent a small proportion of the possible combinations of splice variants and β subunits.

However, it is important to note that these data are only a proof in principle that β subunits might selectively interact with splice variants of the α subunits. We have recently shown that the effects of several β subunits on gating of a voltage-gated sodium channel are obscured at higher temperatures (see table 2 in [19]), and as we were most interested in dissecting the differences between a representative β subunit and the splice variants of *SCN9A* we carried out recordings at room temperature to maximise the sensitivity of detection. Moreover, the full function of β subunits is likely to be dependent on cellular background with many key β subunit functions appearing to be restricted to neurons [20], thus in order to determine the true functional importance of the different interactions, recordings would have to be done in a neuronal setting where the full functional roles of β subunits can be probed. Our data indicate that these recordings will require some means of assessing which splice variants of α subunits are present prior to limiting β subunit.

### Implications for SCN9A

We chose *SCN9A* because this encodes one of the sodium channel α subunits which has the most carefully aligned genotype/phenotype relationship [11]. For this channel it is known that relatively small shifts in current activation are sufficient to lead to clinically important effects. While caution is required in translating between currents seen in HEK cells and those expected in neurons, it is possible that small changes imposed by the co-expression of β subunits could have clinically significant effects. Recent work in β1 knockout mice showing significant effects on the voltage-dependence of inactivation of TTX-sensitive currents in DRG neurons suggests β1 subunits are present in these cells and may modify Na_V_1.7 inactivation, albeit in the opposite direction seen in HEK cells [21]. Mutations in *SCN9A* which hyperpolarise Na_V_1.7 activation (see e.g. [22], [23], [24]), or cause a gain of function by impairing fast-inactivation of Na_V_1.7 channels (see e.g. [15], [25]), can both cause diseases of pain in humans. It has already been suggested that splicing in *SCN9A* may alter the severity mutations in the gene [25], and our data suggest that accessory subunits may also contribute to functional differences between variants. Healthy human DRG express a mixture of all four Na_V_1.7 splice variants, however the expression pattern has been shown to change in an animal model of nerve injury [7]. Our data suggest a role for accessory subunits in determining the consequences of these changes in splicing. This role may also be subject to regulation, because β1 subunits themselves are dynamically regulated during development [9], [26] and disease, including spinal cord injury [27].

### Splicing Combines with Accessory Subunits to Uncouple Modulation of Activation and Inactivation

It is surprising that the presence of the β1 subunit can significantly influence two different functional parameters for the two Na_V_1.7 splice variants, and suggest that in this system different regions of the channel may interact with the β1 subunits. Theeffects of β1 on inactivation in constructs containing the long form of exon 11 imply that there may be some interaction between β1 and the intracellular loop between domains I and II in Na_V_1.7 that contributes to the regulation of inactivation. This loop is partially coded for by exon 11 and is extended in length in the 11 L splice variant by 11 amino acids [6]. The altered activation of channels associated with splicing of exon 5 in the presence of β1 subunits suggests the S3–S4 linker of the first domain and the extracellular domain of the β1 subunits may also interact. Splicing at exon 5 consistently alters a single evolutionarily conserved charged amino acid in this short extracellular linker in the α subunit of several sodium channels [1], and it is possible this amino acid contributes to the altered interaction with β1. In the related Na_V_1.1 subunit, we found increasing temperature reduced the effects of β subunits [19]; this may indicate that the interactions seen in HEK cells are less stable than those in neurons.

This study provides the first evidence that evolutionary conserved splice variants of a voltage-gated sodium channel, in this case Na_V_1.7, can interact in a functionally distinct fashion with sodium channel accessory subunits. The consequences of this interaction in neurons remains to be determined; this study nonetheless demonstrates a regulatory mechanism for sodium channel function dependent on accessory subunit interactions that may be relevant to the whole nervous system.
